# IS LONELINESS CONTAGIOUS? AN ECOLOGICAL MOMENTARY ASSESSMENT WITHIN ROMANTIC COUPLES

**DOI:** 10.1093/geroni/igad104.1416

**Published:** 2023-12-21

**Authors:** Martina Luchetti, Fiona Rupprecht, Jana Nikitin, Alyssa Gamaldo, Jacqueline Mogle, Thomas Ledermann, Martin Sliwinski, Angelina Sutin

**Affiliations:** Florida State University, College of Medicine, Tallahassee, Florida, United States; University of Vienna, Vienna, Wien, Austria; University of Vienna, Vienna, Wien, Austria; Clemson University, Clemson, South Carolina, United States; Clemson University, Clemson, South Carolina, United States; Florida State University, Tallahassee, Florida, United States; Pennsylvania State University, State College, Pennsylvania, United States; Pennsylvania State University, State College, Pennsylvania, United States

## Abstract

Although marriage has been found to be protective against feeling lonely, loneliness might also be ‘contagious’ and spread from one individual to another, particularly within close relationships. Feelings of loneliness tend to correlate within spouses and are associated with relationship strain and lower perceived support from the partner. Despite interest in loneliness among married couples, little is known about how loneliness might affect romantic partners, especially in everyday life. In this study, we examined momentary states of loneliness within middle-aged and older couples, and how these states affect each partner over the course of days. Data were from 137 opposite-sex couples (274 participants), between 40 and 70 years old, who completed phone-based assessments of loneliness on a scale from 0to 100, three times a day, for eight consecutive days. Data were modeled using a combination of dynamic structural equation modeling and actor partner interdependence modeling. For both couple members, auto-regressive associations indicated that loneliness states carried over from moment to moment for both partners. In addition, loneliness of the female partner at time t was associated with loneliness of the male partner at the previous assessment (time t - 1), but not vice versa. Partner effects were in part moderated by relationship quality (strain, perceived support). The results provide insight into how loneliness functions within couples in everyday life contexts and suggest that loneliness might have dyadic implications for the health and well-being of aging couples.

